# Assigning the unassigned: A signature-based classification of rDNA metabarcodes reveals new deep-sea diversity

**DOI:** 10.1371/journal.pone.0298440

**Published:** 2024-02-29

**Authors:** Inès Barrenechea Angeles, Ngoc-Loi Nguyen, Mattia Greco, Koh Siang Tan, Jan Pawlowski

**Affiliations:** 1 Department of Earth Sciences, University of Geneva, Geneva, Switzerland; 2 Department of Genetics and Evolution, University of Geneva, Geneva, Switzerland; 3 Department of Geosciences, UiT-The Arctic University of Norway, Tromsø, Norway; 4 Institute of Oceanology, Polish Academy of Sciences, Sopot, Poland; 5 Institute of Marine Sciences, Spanish National Research Council, Barcelona, Spain; 6 Tropical Marine Science Institute, National University of Singapore, Singapore, Singapore; 7 ID-Gene Ecodiagnostics Ltd., Plan-les-Ouates, Switzerland; Universita degli Studi di Urbino Carlo Bo, ITALY

## Abstract

Environmental DNA metabarcoding reveals a vast genetic diversity of marine eukaryotes. Yet, most of the metabarcoding data remain unassigned due to the paucity of reference databases. This is particularly true for the deep-sea meiofauna and eukaryotic microbiota, whose hidden diversity is largely unexplored. Here, we tackle this issue by using unique DNA signatures to classify unknown metabarcodes assigned to deep-sea foraminifera. We analyzed metabarcoding data obtained from 311 deep-sea sediment samples collected in the Clarion-Clipperton Fracture Zone, an area of potential polymetallic nodule exploitation in the Eastern Pacific Ocean. Using the signatures designed in the 37F hypervariable region of the 18S rRNA gene, we were able to classify 802 unassigned metabarcodes into 61 novel lineages, which have been placed in 27 phylogenetic clades. The comparison of new lineages with other foraminiferal datasets shows that most novel lineages are widely distributed in the deep sea. Five lineages are also present in the shallow-water datasets; however, phylogenetic analysis of these lineages separates deep-sea and shallow-water metabarcodes except in one case. While the signature-based classification does not solve the problem of gaps in reference databases, this taxonomy-free approach provides insight into the distribution and ecology of deep-sea species represented by unassigned metabarcodes, which could be useful in future applications of metabarcoding for environmental monitoring.

## Introduction

The past decade has seen environmental DNA (*e*DNA) metabarcoding become a common tool to assess biodiversity, with the capacity to overcome the limitations of traditional morphology-based methods. Yet, the taxonomic assignment of metabarcoding data remains problematic mainly due to the paucity of reference databases [1,2]. The problem concerns generally the surveys of prokaryotic communities, which are dominated by unknown taxa, also called “microbial dark matter” [3] especially in extreme environments [4,5], polar [6], deep-sea [5] and hydrothermal vents [4,5]. However, the unassigned sequences also prevail among protist and meiofaunal communities [7–9]. These sequences are commonly lumped into an assemblage of unassigned or unknown metabarcodes. Lacking taxonomic information, these sequences cannot be included in biodiversity or biogeography assessments, except as "unknown". Different strategies have been proposed to overcome this problem. A recent study showed that taxonomic assignment approaches based on sequence similarity and composition outperformed more complex phylogenetic and probabilistic methods [10], the accuracy of taxonomic assignment based on the percentage similarity of short metabarcodes is generally low such as in 18S rRNA gene [11] and TrnL P6 loop [12]. Alternatively, a network approach was proposed to characterize unknown species and elucidate their relationships [5].

Here, we tackle this issue by classifying the unassigned metabarcodes into novel lineages using an ultra-short nucleotide sequence that can distinguish one lineage from another, called DNA signatures or signatures character. In general, a DNA signature has focused on single genes (e.g., 16S/18S rDNA gene, mitochondrial COI gene), and could be selected by using sequence alignments in the conserved gene regions. DNA signatures of closely related species or close phylogenetic lineages are expected to be more similar to one another. The signature-based approach to detect and identify microorganisms has been proposed already earlier [13,14], yet its use in current prokaryotic taxonomy is relatively limited since number of sequenced genomes has continued to increase dramatically [15]. This approach is useful in the case of eukaryotes, whose genomic reconstruction is limited compared to prokaryotes [16]. Among eukaryotes, distinctive molecular patterns are generally used to resolve the taxonomy of closely related species [17] or to analyze geographic patterns [18]. A recent study demonstrated the usefulness of DNA signatures to facilitate the taxonomic identification of ciliated protists [18]. Therefore, the nuclear and mitochondrial genes of a microbial eukaryote may bear the signatures needed to integrate both phylogenetic and ecological information.

In our study, we applied the DNA signatures to classify deep-sea unassigned benthic foraminiferal sequences. The recent global metabarcoding analysis showed that the diversity of deep-sea benthic eukaryotes is huge and by far exceeds that of species living in surface waters [19]. However, due to the remoteness of deep-sea habitat, our knowledge about its biodiversity is limited and the majority of eukaryotic metabarcodes obtained from deep-sea sediment DNA remain unassigned. This concerns not only microbial eukaryotes but also metazoan meiofauna, which abound in deep-sea sediments [20]. Unsurprisingly, the metabarcoding surveys reporting the composition of deep-sea microbial and meiofaunal communities are dominated by unassigned taxa.

We focused on foraminifera, which comprises a significant fraction of deep-sea benthic diversity [21–23] and represents more than 50% of the total biomass in Clarion Clipperton Fracture Zone [21], Antarctic Peninsula [24], hypoxic and anoxic environments [21,25]. It has been suggested that at least some deep-sea foraminiferal species are distributed globally based on ribosomal DNA barcodes of isolated specimens [26,27]. This has been confirmed by studies reporting several cosmopolitan foraminiferal amplicon sequence variants (ASVs) or operational taxonomic units (OTUs) in deep-sea metabarcoding data [28,29]. Yet, most of these globally distributed metabarcodes could not be assigned or have only been assigned at higher levels (class, order). According to some studies, the proportion of unassigned sequences in the deep-sea foraminiferal datasets exceeds 50% [28,29].

The material for this study comes from the Eastern Pacific’s Clarion-Clipperton Fracture Zone (CCFZ), an area of potential polymetallic nodule exploitation. The biological community of CCFZ was targeted by several biodiversity surveys [30–32]. The foraminiferal assemblage of CCFZ was shown to be dominated by monothalamous taxa, most of which remained morphologically and genetically unidentified [29,33,34]. We performed a metabarcoding analysis on sediments across different areas of CCFZ and characterized the foraminiferal metabarcodes, focusing on those that were unassigned. We classified them into 61 new lineages, each defined by specific signatures in the hypervariable region of the 18S rRNA gene. We then compared the lineages from CCFZ with other deep-sea basins and shallow-water regions. The taxonomy of the new lineages and their potential use for environmental monitoring of deep-sea resources are discussed.

## Material and methods

### Sediment sample collection

The sampling was carried out within the contract area assigned to Ocean Mineral Singapore by the International Seabed Authority. In this study, 36 samples were collected in 2020 using 1mx1m box cores during RESOURCE Cruise 01 (OMS license area). At each station, three replicates were taken with a 50 ml sterile syringe with the end cut off. The syringe was inserted into the sediment in order to collect at least 5 cm. As we were interested only in the surface sediments, we pushed the sediment lengthwise into a plastic cup where the last centimeters were discarded. Only the first 1–2 centimeters were placed into a tube with 10 ml of LifeGuard Preservation solution (Qiagen, Germany). Samples were frozen on board, shipped frozen to the University of Geneva, and stored at -20°C until their extraction.

### Sediment DNA extraction, amplification, and sequencing

The sediment samples were extracted using the manufacturer’s guidelines of the DNeasy® PowerMax® Soil Kit (Qiagen, Germany). To target foraminifera eDNA, the 37F hypervariable region of the nuclear 18S rRNA gene (68–196 bp), was PCR amplified using specific primers [27]. To allow multiplexing of samples in one library, the forward s14F1 5′-AAGGGCACCACAAGAACGC-3′ and reverse s15 5’- CCACCTATCACAYAATCATG-3’ primers were tagged with unique 8 nucleotides at the 5’ end [35]. Three PCR replicates were amplified and pooled for each sample before being quantified using high-resolution capillary electrophoresis (QIAxcel System, Qiagen, Germany). The PCR products were pooled in equimolar concentration. Dimers and short amplicons (< 100 bp) were then excluded from the pool using the High Pure PCR Product Purification Kit (Roche), as the shortest amplicon including the primers and tags is 123 bp. The library was prepared using TruSeq® DNA PCR-Free Library Preparation Kit (Illumina, USA), and its concentration was quantified using Kapa Library Quantification Kit for Illumina Platforms (KAPA Biosystems, USA). Finally, the library was sequenced with a MiSeq instrument using paired-end sequencing for 300 cycles with a v.2 kit.

### Bioinformatics analysis

We combined the obtained sequence with the published ones from other sites from CCFZ, and other deep-sea foraminifera datasets obtained from samples between -4000 and -9000 meters of water depth from the North Atlantic, Mid Atlantic, South Atlantic, Southern Ocean, and Northwest Pacific [29,36] (see S1 Table), and available in ENA under the following accession number PRJEB44134, PRJNA554310, and PRJNA899048. We also added the shallow water foraminifera datasets from the Tyrrhenian Sea [37], Adriatic Sea [38–41] and around Svalbard [42] (see S1 Fig), available under the following accession numbers: PRJNA723313, PRJNA897836, PRJNA813562, PRJEB29469, and PRJNA768352. Some of those datasets were obtained using primers s14F1- s17 [43] and therefore targeting two hypervariable regions of 18S (37F and 41F), including the studied region.

The raw datasets were processed using the SLIM software [44]. First, they were demultiplexed and the primers were removed using the module *demultiplexer*. The paired fastq files from all datasets were combined and processed together (quality filtering, denoising, merging, and chimera removal on sequences) using the module DADA2 [45] implemented in SLIM. The DADA workflow was set to default parameters, without length truncation and pseudo-pooling as the pooling parameter for the inference of ASV. Then, we clustered the obtained Amplicon Sequencing Variants (ASVs) at 97% similarity into OTUs and continued with a LULU curation [46] as recommended in [47]. This curation removes erroneous clusters coming from intra-individual variability or errors during PCR or sequencing. The clustering at 97% was done using the DECIPHER R package and the curation with the LULU R package with the default parameters.

To retain only foraminifera sequences obtained with s14F1 -s15 primers, we identified conservative motifs across all foraminiferal species in the region 37 flanking the hypervariable region, i.e., before the beginning of 37F and at the end. Using *grep* command in R or bash we removed sequences not having “GACAG”, adjacent to the foraminiferal-specific hypervariable region 37F [27] and at the end of the 37 conservative region “TAGTCCTTT” and “TAGTCCCTT”. In some species, we noticed the presence of substitution (T > C) therefore we used these two patterns. The remaining sequences were then filtered by their size and abundance, we retained sequences with > 70 bp and > 100 reads.

Some shallow-water sequences were obtained using the primer pairs s14F1- s17 covering the 37f and 41f variable regions. For them, we retained sequences only if they contained “GACAG” in the 37 region and “GGTGGT” in the 38 conserved region.

We used three probabilistic approaches to assign the sequences taxonomically and to identify the unassigned sequences: VSEARCH [48] at 95% similarity, IDTAXA [49] at 60% of confidence, and BLAST+ [50] at 95% similarity and 100–99% of coverage. We used our local database of benthic foraminifera including selected sequences from GenBank and the planktonic foraminifera ribosomal reference database—PFR2 [51]. The resulting 4602 reference sequences cover Globothalamea, Tubothalamea, and the paraphyletic groups of monothalamids. The monothalamids comprised well-defined clades (e.g., Clade A [52]), the ENFOR (ENvironmental FORaminifera) groups consisting of environmental clades from previous metabarcoding studies obtained through cloning and Sanger sequencing (e.g., ENFOR1 [53]), and/or poorly defined clades (e.g., Monothalamids X or undetermined Monothalamids), comprising mainly the so-called squatter species [54,55].

### DNA signature identification

We prepared a subset of the CCFZ dataset including 2245 OTUs that could not be assigned by VSEARCH as well as those that VSEARCH assigned to ENFOR or Monothalamids X. All sequences with more than 2–3 deletions, insertions, or ambiguities in the conserved regions located before the highly variable region 37F were removed, as we assumed that the conserved regions should contain similar sequences across all foraminiferal OTUs. Sequences having similar molecular signatures at the beginning or the end of the 37F region were regrouped into lineages. The signatures were validated if the number of reads was superior to 5000 reads and the lineages comprised at least 2 OTUs. The retained lineages were compared with the annotations made previously. Lineages were not considered if the signature recognized a group already present in the database, except if they were assigned to an environmental clade or a Monothalamids X. After these restrictive filters, only 693 OTUs were used to define the unique signature, corresponding to each lineage. The remained lineages were named by the letter L and a number (e.g., L1, L43). A letter was added after the number (e.g., L2A, L2B) to differentiate similar lineages sharing most of the characters, thus obtaining sub-lineages. We produced an R script, available on GitHub (https://github.com/MatGreco90/ForamSignature), with the *biostrings* package, which allowed identifying the patterns without a mismatch in CCFZ, deep-sea and shallow water datasets. The relative abundance was calculated using the *make_relative* function within the *funrar* package while the map was drawn using the following libraries *rnaturalearth*, *rnaturalearthdata*, and *ggspatial*.

### Phylogenetic analysis

Phylogenetic tree specific to new lineages was constructed, covering the entire monothalamids to assign taxonomy and resolve undescribed clades. A total of 693 OTUs of new lineages and 388 reference sequences from well-described monothalamids were included in the phylogenetic tree construction. As an outgroup, we used two sequences from non-foraminiferal rhizarians (*Cercomonas longicauda* and *Gromia oviformis*). We aligned our sequences using the E-INS-i iterative refinement method in MAFFT v.7 [56]. Trees were built using the IQ-TREE maximum likelihood method [57,58]. Ultra-fast bootstrapping [59] was used to generate branch support values with 1000 bootstrap replicates. Phylogenetic tree visualization and annotation were done using the R package *ggtree* v.1.12.7 [60]. Default alignment parameters were used to align and generate a phylogenetic tree. Based on the phylogenetic tree, the 43 lineages were grouped into 27 higher-ranking groups (e.g., CCZ1). This provides an appropriate degree of phylogenetic specificity for each signature (S4 Table).

## Results

### Sequence data

After the clustering, LULU curation, removal of non-foraminiferal sequences, and a filter of rare ASV (< 100 reads) the CCFZ dataset contained 37,127,019 reads and 2382 OTUs, the other deep-sea areas dataset 48,559,807 reads corresponding to 4148 OTUs and the shallow water dataset comprised 26,349,529 reads and 3745 OTUs. Details of the number of reads retained at each step and for each basin are detailed in S1 Table.

### Taxonomic assignment

At first, the OTUs were assigned using the three standard methods, i.e., VSEARCH, BLAST, and IDTAXA. All three methods recognized the main groups of foraminifera: globothalamids, tubothalamids, and monothalamids. However, less than 50% of OTUs were assigned. VSEARCH assigned the greatest fractions of sequences (46.2%), followed by BLAST (24.1%) and IDTAXA (10.2%). The monothalamids, including environmental sequences (ENFOR) and Monothalamids X, were the most abundant groups of foraminifera (S1 Fig, more details in S3 Table). Globothalamids and tubothalamids were the minority in the three assignments. According to the VSEARCH assignment, globothalamids and tubothalamids made up roughly 4.9% (561,586) of reads, monothalamids, including ENFOR and Monothalamids X, represented 41.28% (5,554,157) of reads, while unassigned OTUs accounted for 53.73% (21,466,294 reads).

**Fig 1:**
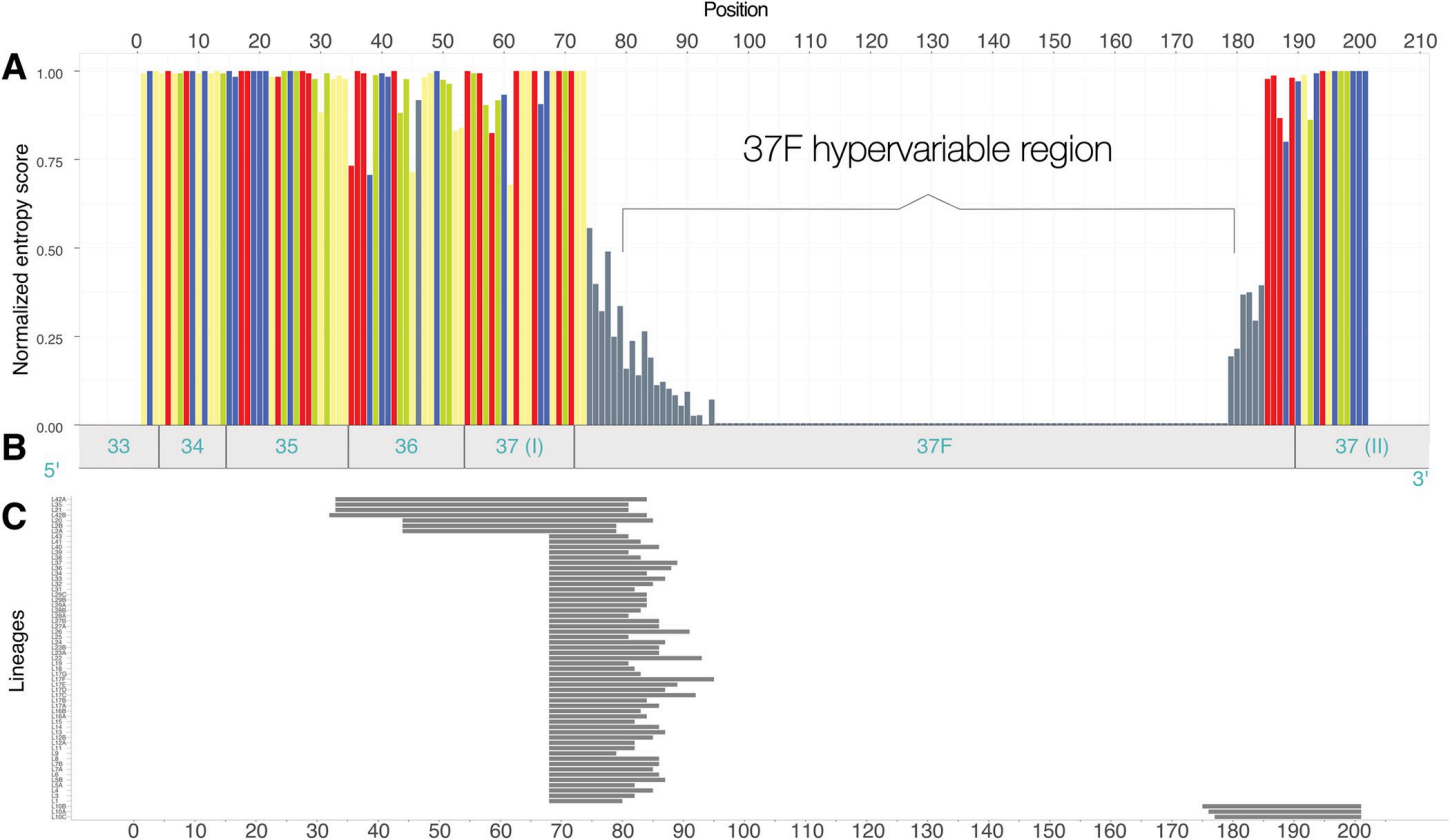
Positions of signatures in the foraminiferal 18S rRNA gene. (A) entropy plot and (B) foraminiferal regions from 33 to 37 after [27], (C) position and length of signatures.

From sequence alignment of 693 unassigned OTUs, a total of 61 DNA signatures were identified corresponding to 30 lineages and 31 sub-lineages (S4 Table). The length of signatures varied between 12 and 53 nucleotides. Most of the signatures (51) were located at the beginning of the 37F variable region, comprising the six conservative nucleotides “GACAGG” at the end of the 37 (I) helix (Fig 1). Seven signatures started in the 35 or 36 regions and finished in the 37F variable region. We also used the end of 37F and 37 (II) regions to discriminate three sub-lineages (Fig 1).

**Fig 2:**
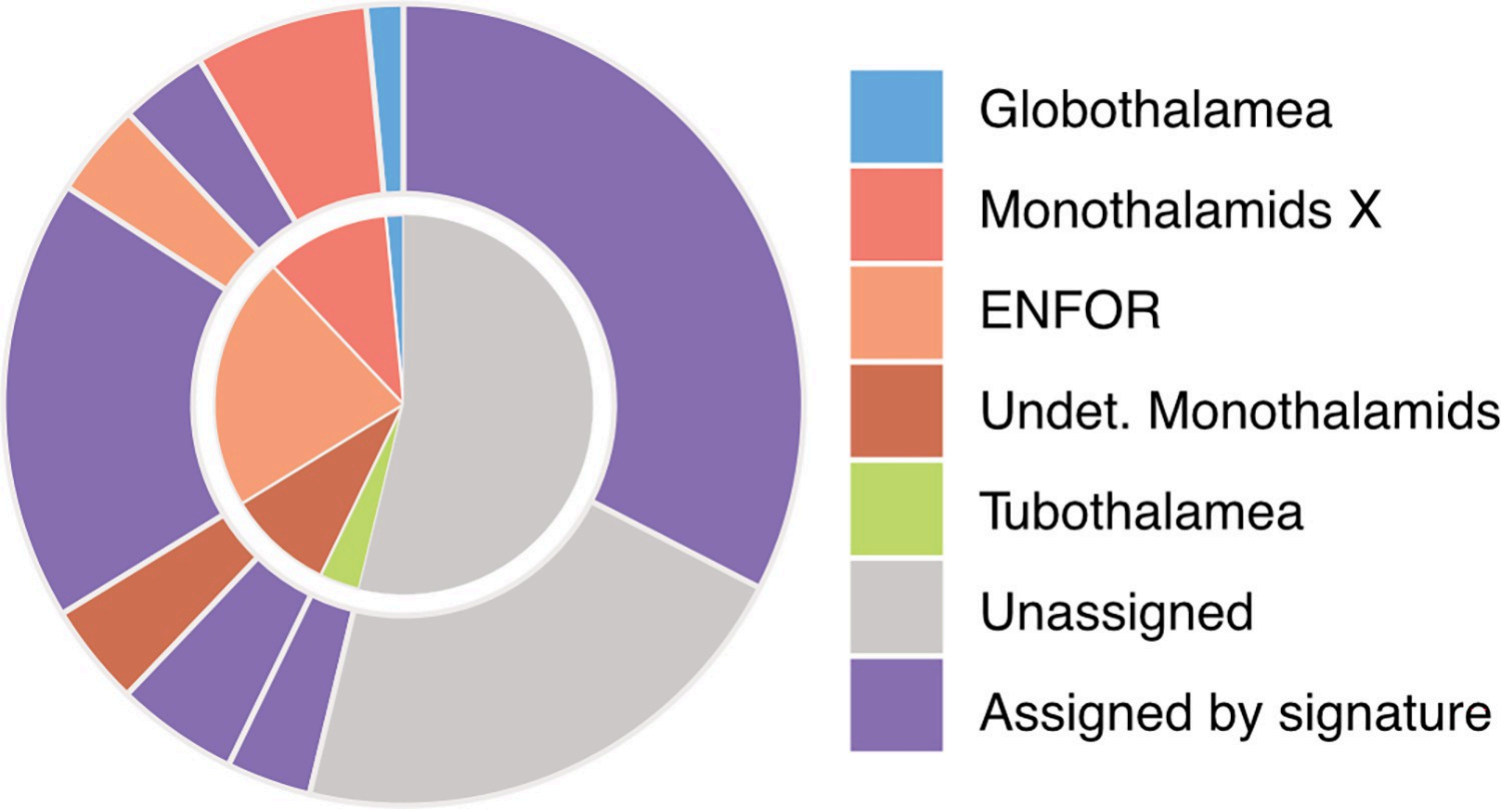
The pie chart shows the proportion of foraminifera groups before and after being assigned by signatures. The inner pie chart represents the result of VSEARCH assignments, and the outer ring represents a combined assignment including VSEARCH and signature-based approach (in purple). The foraminiferal groups are assigned by signatures including the new lineages in unassigned, monothalamids, ENFOR, and other (undetermined) monothalamids.

By searching for the signatures without a mismatch (i.e., with 100% similarity), we could identify 109 additional OTUs in the CCZ dataset (see S5 Table). In total, 802 OTUs (corresponding to 34% of the total number of OTUs and 62% of the total number of reads) were assigned to novel lineages. The signature approach allowed to reduce the number of unassigned OTUs to 21% (Fig 2). The signatures were also found in many sequences already identified with VSEARCH at 95% similarity. The largest proportion of OTUs included in new lineages (82%) was found among the environmental ENFOR clades. We also found a large proportion of OTUs assigned to novel lineages among the monothalamids (34%) and the undetermined monothalamids (Monothalamids X, 54%). One of the novel lineages (L21) was assigned to both monothalamids and tubothalamids, but this requires confirmation by single-cell sequencing. No signature was found among globothalamid sequences.

### Phylogenetic placement of new lineages: definition of new clades

**Fig 3:**
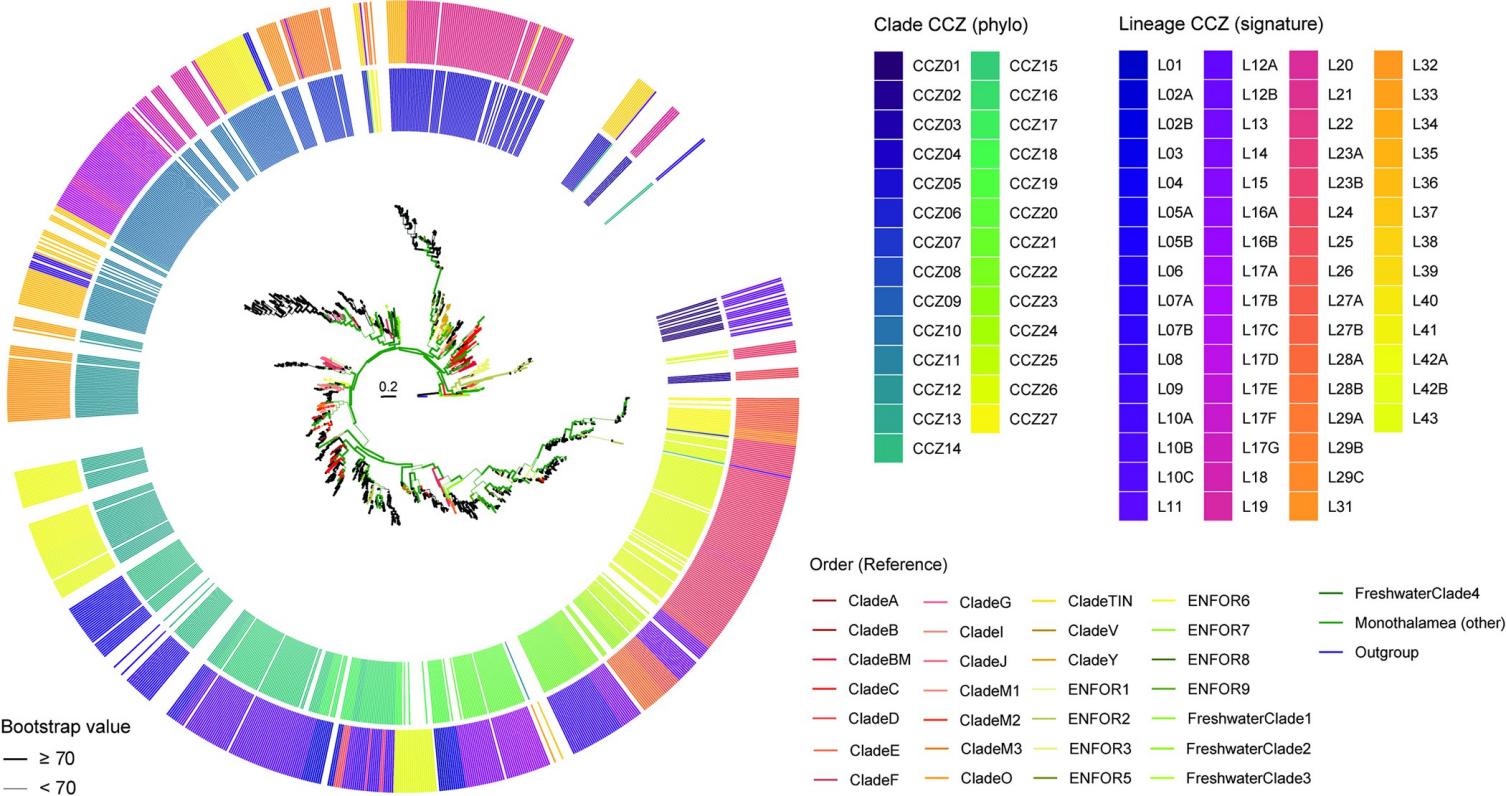
Phylogenetic diversity and novelty of foraminiferal OTUs identified by signatures. Phylogenetic analysis of selected OTUs representing new lineages and reference sequences of monothalamids from Clade A to Clade Y and some freshwater clades. Tree branches are colored at the Order level. All sequences were aligned with MAFFT, and trees were constructed with IQ-TREE, based on the GTR+F0 model of evolution with 1000 bootstrap replicates. Bold branches indicate ≥ 70% bootstrap support. Scale bars are in units of substitutions per site. The rings indicate clusters based on phylogenetic position (inner ring) and signatures (outer ring).

To evaluate the taxonomic assignment of the signature-based approach, we constructed a phylogenetic tree from the 693 OTUs containing the signature with reference monothalamid sequences. A simplified version of the tree is presented in Fig 3 with a more detailed version provided in S2 Fig. Most of the new lineages formed monophyletic groups. They belonged to the previously established clades of monothalamids (e.g., Clade C, Clade M, Clade I, Clade V) and environmental DNA‐derived foraminiferal sequences (ENFOR clades).

Phylogenetic analysis indicated that the signatures of the assigned lineages were more similar to each other than to those of distant ones (Figs 3 and S2). Most of the new lineages were placed on the tree at the specific clades, which indicated a general agreement between their signature assignment and phylogenetic positions. Interestingly, some new lineages were found in specific groups that are highly related to other CCFZ sequences from the database (i.e., L14, L19, L21, L23B, L28A, and L42A). The OTUs of one lineage (L17) form a group on their own, with no closest reference-related sequences.

### Biogeography of new lineages

**Fig 4:**
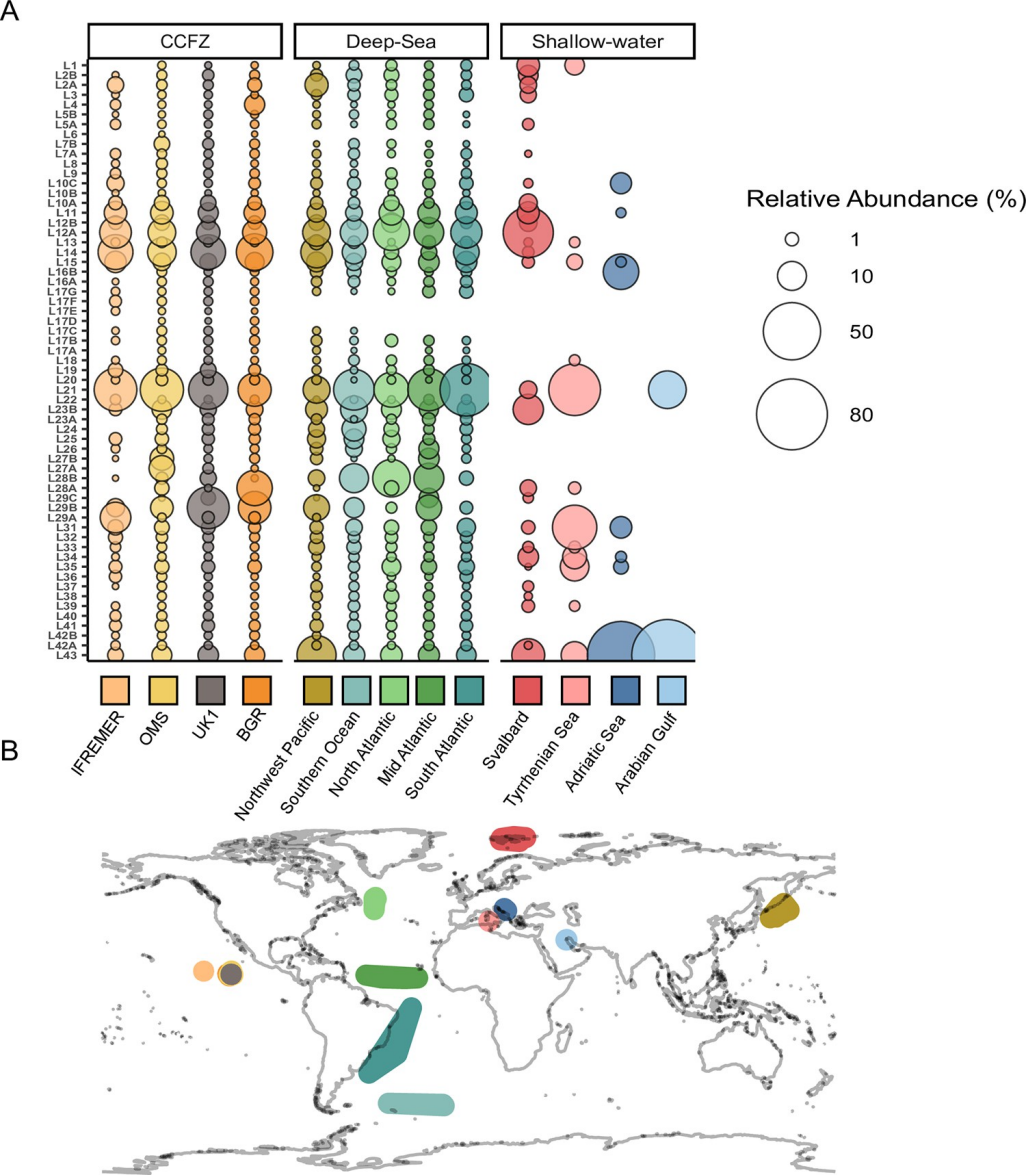
Bubble chart showing the proportions of 61 lineages present and their distribution in the studied regions. (A) The bubble sizes show the relative abundance of lineages per area. The bigger the bubble, the more abundant the lineage is in each area. (B) A map showing CCFZ, other deep-sea areas, and shallow water sites (<200 m depth).

The comparison of metabarcoding datasets within CCFZ and with other deep-sea and shallow-water sites showed clear patterns of distribution of the newly defined lineages (Fig 4). Within the CCFZ, the OMS and UK-1 areas shared all the lineages whereas in BGR he lineage L29Cwas absent. The IFREMER area, located in the westernmost part of CFFZ, has the lowest number of lineages (49) shared with the eastern part of CCFZ sites. Comparing CCFZ to deep-sea sites, 85% of lineages were the most deep-sea regions. Only five lineages were endemic to CCFZ (absent in all other areas): L6, L17D, E, F, and 27A. 56 lineages occurred in the Northwest Pacific, 53 in the Southern Ocean, and 50 in the three regions of the Atlantic Ocean. Lineages 28A and 29C only appeared in the North Atlantic and the mid-Atlantic, respectively. L4 was present in the North and mid-Atlantic and L8 and L17A were found in the mid and south of Atlantic.

Compared to the deep-sea, 30 out of 61 lineages were also present in shallow-water sites. 26 lineages were present in the Arctic fjords (Svalbard), while 10 were found in the two Mediterranean Sea sites. Only five lineages were present globally, including the Persian Gulf. Two of them (L21, L43) were the most abundant and had in common with the other three cosmopolitan lineages a very short signature.

**Fig 5:**
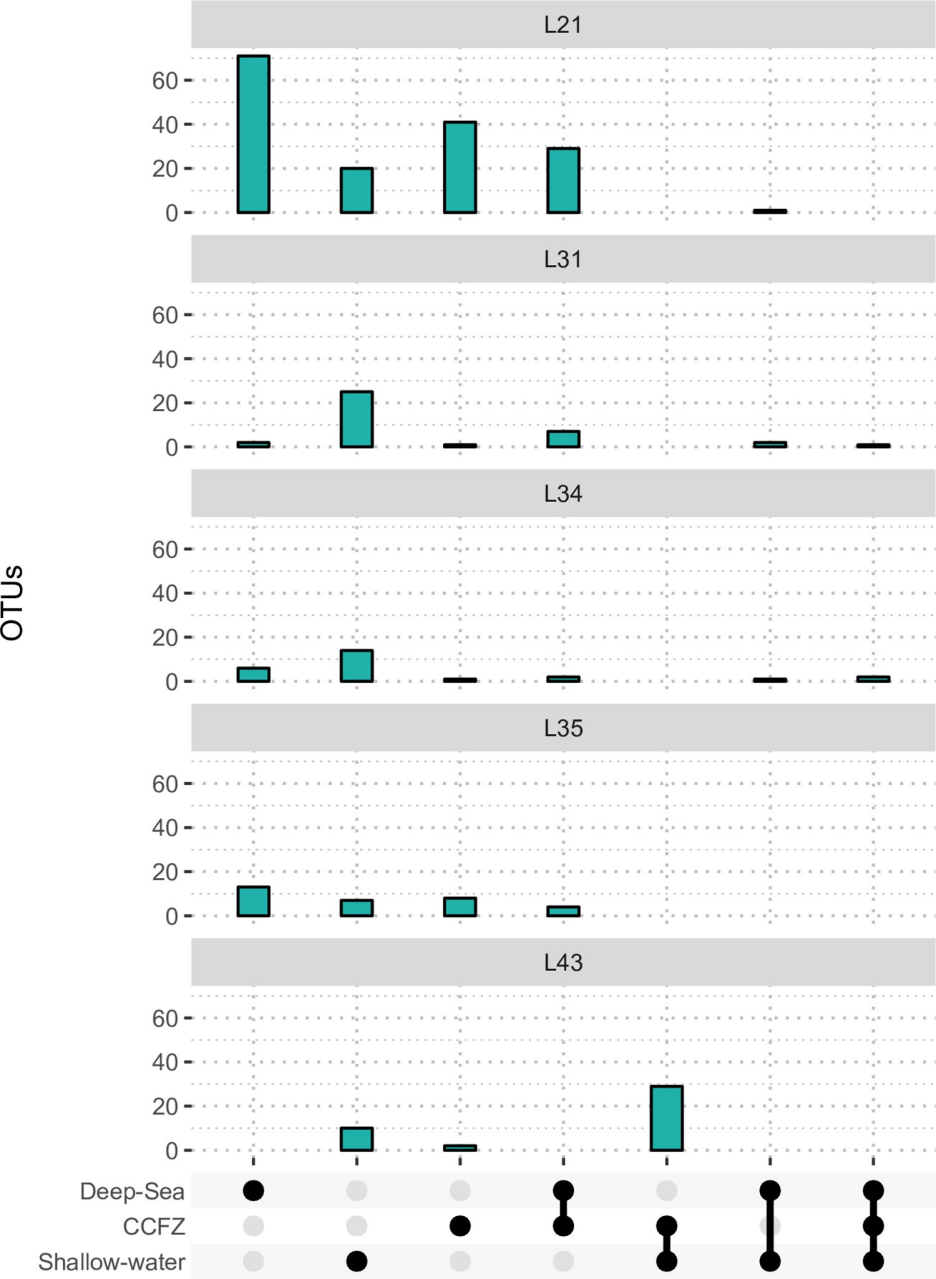
UpSet chart showing the five most abundant lineages. It comprises OTUs shared between CCFZ, deep-sea, and shallow-water samples. All duplicate OTUs were removed and the number of OTUs is a conservative estimate per habitat.

To better understand the biogeography of the five cosmopolitan lineages (L21, L31, L34, L35, and L43), we analyzed the distribution of OTUs composing these lineages. The highest diversity in terms of the number of OTUs retrieved was observed in L21, which counted a total of 162 OTUs. Most of the OTUs were characteristic of deep-sea sites (71), with 41 OTUs exclusive to CCFZ sites, while 29 were shared between them (Fig 5). Within this lineage only a single OTU occurring in the shallow-water datasets was also observed in the deep-sea.

The lineages L31 and L34 presented an overall lower diversity in terms of OTUs’ number (38 and 26 OTUs respectively), with the majority of the OTUs retrieved uniquely from shallow water samples. Along with L43, L31, and L34 were the only three lineages presenting OTUs with a distribution encompassing all the ecosystems analyzed. In particular, the overall diversity of L43 constituted 63% of OTUs occurring in all the datasets. In contrast, L35 mainly presented OTUs with habitat-specific distributions with only 5 OTUs shared between CCFZ and deep-sea sites.

## Discussion

Despite the advances introduced by metabarcoding, taxonomically unassigned sequences remain an issue for researchers interested in biological diversity assessment and ecology. As shown by our study, about half of the deep-sea metabarcodes could not be assigned. This proportion is even higher if we also consider as unassigned the metabarcodes that were classified only at higher levels (phylum or class). Indeed, the assignment at such a high taxonomic level provides no information about the biology of organisms represented by given sequences, ASVs, or OTUs, hampering any attempt of their ecological interpretation.

By using diagnostic 18S rDNA signatures, we were able to increase the number of assigned reads to 54% when using bioinformatics tools (VSEARCH, IDTAXA, and BLAST) to 80% using the signature approach (Fig 2). In total, 61 new foraminiferal lineages have been defined based on DNA signatures. As expected, most of these lineages belong to monothalamids, a paraphyletic assemblage of early-evolved single-chambered foraminifera [52], which are generally overlooked in conventional foraminiferal surveys [61]. Our study confirms the importance of this group in the deep-sea environment [21] and provides a general scaffold for its classification.

Besides this taxonomic aspect, our approach can also contribute to a better understanding of the ecology and geographic distribution of deep-sea foraminifera. This information could be lost if the unassigned foraminiferal sequences are lumped together. Some authors analyzed metabarcoding data at the level of ASV or OTU, for example, in the study of patchiness of deep-sea foraminifera [62] or their distribution along the depth gradient [36] or even in coastal biomonitoring [63]. Yet, the ASV or OTUs represent a very low taxonomic level, corresponding to species or intraspecific variants. Inferring general patterns of distributions and ecological adaptations based on foraminiferal ASVs or OTUs might be difficult, especially given the presence of intragenomic polymorphism in this group [64]. By classifying ASV/OTUs at higher taxonomic levels our approach facilitates their correlation with environmental variables.

The advantages of this approach are well illustrated by the results of our investigation on the distribution of deep-sea foraminifera. Previous studies suggested that some deep-sea species are globally distributed [28,65]. However, the species targeted by these studies (e.g., *Epistominella exigua*) represented genera that are widely distributed in the coastal environment, and the deep-sea species were considered as possessing special adaptations to this particular environment. Our study demonstrates that the numerous foraminiferal lineages are specifically deep-sea. It is well documented that the giant monothalamous foraminifera belonging to Xenophyophorea occur exclusively on abyssal plains [66]. Nevertheless, according to our study, the number of foraminiferal lineages adapted to the deep sea might be much higher than expected.

Admittedly, the signature-based approach does not allow us to exactly determine the taxonomic status of the new lineages. We expect that at least some of them correspond to the genus or species level. This could be the case of lineages specific to CCFZ (L17D, E, F), characterized by a long signature. Our approach is based on the observation that the variability increases progressively at the end of 37 helix and the beginning of 37F variable region [35,67]. Thus, the longer signatures might better define the lower taxonomic level and can reduce the risk of misidentification as in the case of L21, a short signature whose assignment and placement were within monothalamids and tubothalamids species. However, any inference of taxonomic status from a single variable region needs to be treated with caution, given the high variability of evolutionary rates in foraminiferal ribosomal genes [68].

Furthermore, not all foraminiferal species can be distinguished in this region, 37f, as shown by [69] where it was not possible to discriminate Cibicidoides species. This can be solved by increasing the number of metabarcodes obtained through single-cell analysis. Once a comprehensive database of foraminiferal metabarcodes is established, one would have to develop a further signature-based approach to make it useful for taxonomical and ecological studies.

A practical advantage of our approach is its technical simplicity and unambiguity. As the signature patterns are defined at 100% similarity, there is no place for any ambiguity regarding lineage identification. This aspect seems particularly important in the case of short (< 100 bp) metabarcodes, where one SNP equals 1% divergence. The shortcoming of such an approach is that the slightest variation in the signature, even one base change, prevents us from including a given OTU in the lineage. However, if we do not apply this rule, the signatures rapidly lose their specificity. Here, we preferred to create two or more lineages (e.g., A and B) that differ by an SNP, rather than accept one SNP change. Nevertheless, well-defined ambiguities could be accepted in the future, especially if their presence is confirmed by single-cell polymorphism analysis.

To conclude, we view our approach as an inclusive tool that allows expanding the information inferred from metabarcoding data to the currently unassigned metabarcodes. We do not view the signature-based classification as a panacea to fill the gaps in the reference database for particular habitats or taxa. There is no doubt that building a comprehensive reference database is essential for biodiversity surveys. Yet, in certain circumstances, this task might be unrealistic. We are convinced that our approach can be very useful in metabarcoding studies dealing with overlooked taxonomic groups and/or poorly explored habitats, such as the deep sea. It can help in the case of DNA-based environmental monitoring that targets particular groups of bioindicators or in paleo-metabarcoding reconstructions of past biodiversity. Its efficiency will certainly increase if the metabarcoding data are combined with single-cell high-throughput barcoding, but this taxonomy-free approach can be viewed as a practical way to uncover hidden information present in hitherto unassigned metabarcoding data.

## Supporting information

S1 Fig Taxonomic composition at class level and relative abundance of assigned and unassigned sequences using the three common methods: VSEARCH, IDTAXA and Blast. All monothalamids sequences, including the environmental sequences (ENFOR) and sequences not regrouped in a clade that are grouped into undetermined Monothalamids (Undet. Monothalamids) are coloured in shades of orange. More details in S3 Table.
(TIFF)

S2 Fig Extended phylogenetic tree of CCFZ monothalamid sequences (ASVXXX) and monothalamids reference sequences (PAWXXX). The tree was constructed using the maximum likelihood method. The size of circles at nodes represents bootstrap support. The first column of CCFZ sequences is the name, the second the clade, and the third the lineage.
(PDF)

S1 Table List of datasets. Features datasets from CCFZ, Deep-Sea and Shallow-water basins and their accession number.
(XLSX)

S2 Table Filtering reads. Number of reads at each step of filtering per dataset and area.
(XLSX)

S3 Table Number of reads per taxonomic method. Distribution of main foraminifera classes depending on the method of taxonomic assignment, only sequences having more than 100 reads were taken into account.
(XLSX)

S4 Table List of lineages and clades. Details Lineages and clades including the signature (sequence), abundance and number of OTUs.
(XLSX)

S5 Table Taxonomic assignment of sequences that could be assigned by the signatures. Other assignment methods such as VSEARCH, IDTAXA and BLAST are also displayed for comparison.
(XLSX)
