# Supplementary figures and images for: Assigning the unassigned: A signature-based classification of rDNA metabarcodes reveals new deep-sea diversity
Source: PLoS One. 2024 Feb 29;19(2):e0298440. doi: 10.1371/journal.pone.0298440 (PMC10903905; doi:10.1371/journal.pone.0298440)

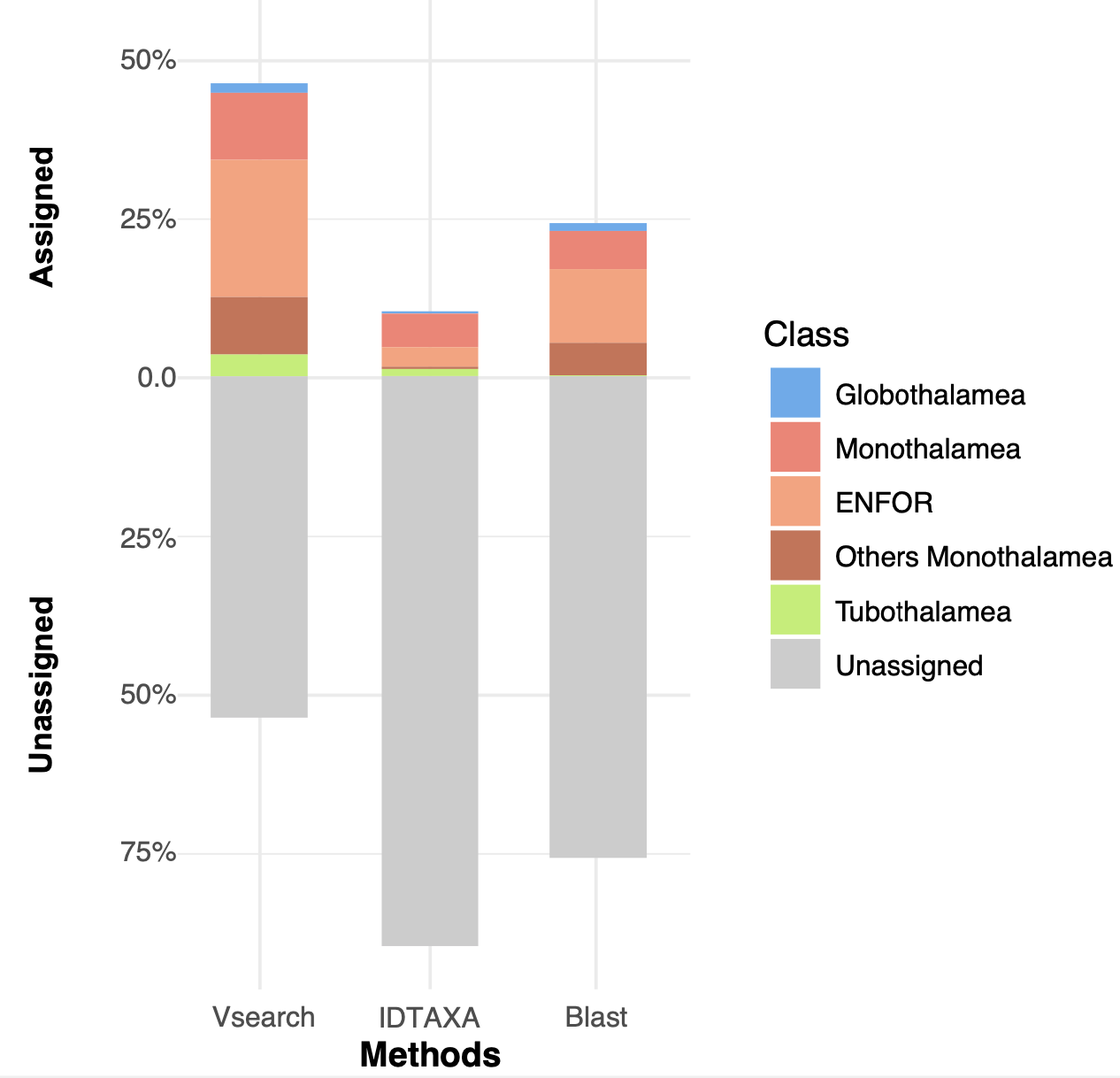

Supplement: S1 Fig — All monothalamids sequences, including the environmental sequences (ENFOR) and sequences not regrouped in a clade that are grouped into undetermined Monothalamids (Undet. Monothalamids) are coloured in shades of orange. More details in S3 Table. (TIFF) [file pone.0298440.s001.tiff]
