# Supplementary material for: Assigning the unassigned: A signature-based classification of rDNA metabarcodes reveals new deep-sea diversity
Source: PLoS One. 2024 Feb 29;19(2):e0298440. doi: 10.1371/journal.pone.0298440 (PMC10903905; doi:10.1371/journal.pone.0298440)

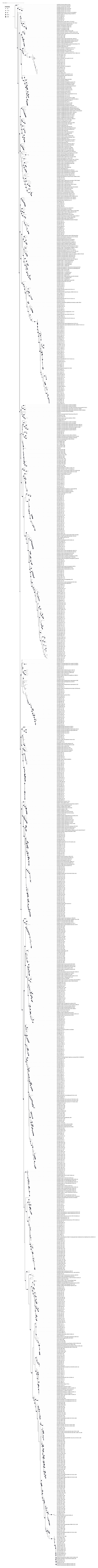

Supplement: S2 Fig — The tree was constructed using the maximum likelihood method. The size of circles at nodes represents bootstrap support. The first column of CCFZ sequences is the name, the second the clade, and the third the lineage. (PDF) [file pone.0298440.s002.pdf]
